# Mineralization of a sulfonated textile dye Reactive Red 31 from simulated wastewater using pellets of *Aspergillus bombycis*

**DOI:** 10.1186/s40643-017-0153-9

**Published:** 2017-05-17

**Authors:** Razia Khan, M. H. Fulekar

**Affiliations:** School of Environment and Sustainable Development, Central University of Gujarat, Gandhinagar, Gujarat 382030 India

**Keywords:** Reactive Red 31, *Aspergillus bombycis*, Pellets, Mineralization, GCMS

## Abstract

**Background:**

Reactive Red 31, applied extensively in the commercial textile industry, is a hazardous and persistent azo dye compound often present in dye manufacturing and textile industrial effluents. *Aspergillus bombycis* strain was isolated from dye contaminated zones of Gujarat Industrial Development Corporation, Vatva, Ahmedabad, India. The decolorization potential was monitored by the decrease in maximum absorption of the dye using UV–visible spectroscopy. Optimization of physicochemical conditions was carried out to achieve maximum decolorization of Reactive Red 31 by fungal pellets.

**Results:**

Pellets of *A. bombycis* strain were found to decolorize this dye (20 mg/L) under aerobic conditions within 12 h. The activity of azoreductase, laccase, phenol oxidase and Manganese peroxidase in fungal culture after decolorization was about 8, 7.5, 19 and 23.7 fold more than before decolorization suggesting that these enzymes might be induced by the addition of Reactive Red 31 dye, and thus results in a higher decolorization. The lab-scale reactor was developed and mineralization of Reactive Red 31 dye by fungal pellets was studied at 6, 12 and 24 h of HRT (hydraulic retention time). At 12 h of HRT, decolorization potential, chemical oxygen demand (COD) and total organic carbon reduction (TOC) was 99.02, 94.19, and 83.97%, respectively, for 20 mg/L of dye concentration.

**Conclusions:**

Dye decolorization potential of *A. bombycis* culture was influenced by several factors such as initial dye concentration, biomass concentration, pH, temperature, and required aerated conditions. Induction of azoreductase, laccase, phenol oxidase, and Mn-peroxidase enzymes was observed during dye decolorization phase. *A. bombycis* pellets showed potential in mineralization of dye in the aerobic reactor system. Isolated fungal strain *A. bombycis* showed better dye decolorization performance in short duration of time (12 h) as compared to other reported fungal cultures.

**Graphical abstract:**
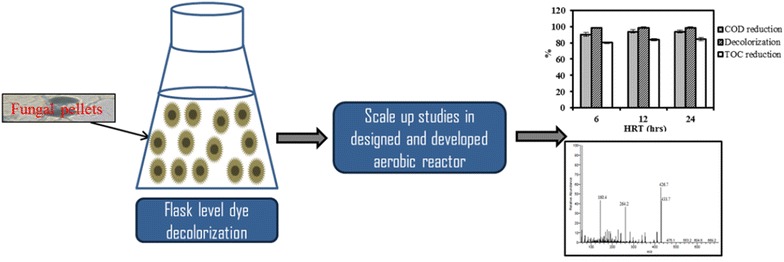
Degradation of RR31 dye in developed aerobic fungal pelleted reactor.

## Background

Industrial wastewater pollution has created a great concern in current world scenario, disturbing ecological balances of the aquatic and terrestrial ecosystem. Dye manufacturing industries are among the most polluting industries critically contaminating water bodies.

Azo dyes represent the largest class of commercial synthetic dyes, with a broad range of colors and represents up to 70% of the total textile dyes utilized (Lang et al. 2013). 280,000 tonnes of textile dyes are discharged worldwide every year in industrial effluents (Jin et al. 2007). Synthetic azo dyes are regularly applied in various textile, leather, food, pharmaceutical, and paper industries (Saratale et al. 2013). The synthetic dyes used in the textile industries have complex aromatic molecular structures making them resistant to biological remediation (Fewson 1988). Dye containing industrial wastewater usually consists of a wide range of organic pollutants including dyes, solvents, and acids which are responsible for eutrophication, O_2_ depletion, and toxicity of receiving waterbodies. Thus, rendering their treatment essential before discharge in waste stream or waterbodies.

Many physicochemical treatment technologies have been applied for the remediation of synthetic dye containing industrial effluents, but possess limitation such as cost effectiveness and often result in the formation of sludge. Biological treatment involving either fungal biomass or their enzymes is proven to be a better alternative. Various fungi possess the ability to degrade complex dye structures by a catalytic mechanism using extracellular enzymes such as azoreductase, laccase, lignin peroxidase, and manganese peroxidase (Gomi et al. 2011). Filamentous fungi represent an attractive agent for dye bioremediation owing to their self-pelletization capabilities. Numerous filamentous fungal strains such as *Aspergillus* sp., *Phanerochaete* sp., and *Basidiomycetes* sp. have shown the phenomenon of self-pelletization (Abd El-Rahim et al. 2016). Application of fungal pellets in remediation of dye containing industrial effluent possesses the advantage of easy harvesting of biomass after treatment and reuse of fungal biomass. Long term operation of bioreactors with fungal pellets under non-sterile conditions is an important area of research requiring greater attention. There are very few studies where the remediation potential of fungal pelleted reactors under non-sterile conditions has been investigated (Nilsson et al. 2006; Tang et al. 2011). Fungal-based dye degradation faces the limitation of being a time-consuming process. As the time required for dye removal are often reported in days. A large amount of colored effluent is released from dye manufacturing and textile industries on a continuous basis. Therefore, there is an urgent need of developing a system involving potent fungal strain possessing ability to degrade dye in short time duration.

The focus of this study is to isolate and identify potent Reactive Red 31 (RR31) dye degrading fungal strain. Effect of various physicochemical conditions on dye decolorization using pellets of potent fungal strain was assessed on flask level experiments. Assay of azoreductase, laccase, tyrosinase, and manganese peroxidase was carried out under optimized conditions. Scale-up studies for RR31 dye degradation using fungal pellets was conducted in a designed and developed aerobic reactor. Mineralization of RR31 dye in the aerobic reactor was determined by conducting COD, TOC and GCMS analysis of treated samples.

## Methods

### Chemicals

All chemicals used in this study were of analytical grade and of the highest purity available. Potato dextrose agar (PDA) and Potato dextrose broth (PDB) were purchased from Hi-Media Laboratories Pvt Ltd (Mumbai, India). All substrates used for enzymatic studies were procured from Merck. A textile dye Reactive Red 31 was obtained from a dye manufacturing industry, GIDC, Vatva, Ahmedabad, India.

### Isolation and screening of dye decolorizing fungal cultures

Isolation of potent dye degrading fungal cultures was carried out from the collected soil samples from various dye-polluted zones of GIDC Vatva, Ahmedabad, and Veraval. Sterilized PDA, amended with 10 mg/L of RR31 dye, was used as screening media. PDA plates were inoculated using serial dilution technique, incubated at 30 °C and monitored after 72 h. The fungal colonies that showed a clear zone of dye decolorization around them were isolated, purified, and maintained on PDA medium. Selection of potent fungal culture was carried out using enrichment culture technique. Each selected fungal strain was inoculated in 250 mL Erlenmeyer flasks containing 100 mL Potato dextrose broth (supplemented with 10 mg/L RR31 dye) and incubated under shaking condition (120 rpm) at 30 °C. After the incubation period, 2 mL aliquots were withdrawn and monitored for dye decolorization as reported by Khan et al. (2014) using Eq. (1);

1 $${\text{Decolorization }}\left( \% \right) \, = \, \left( {\left( {{\text{AC}} - {\text{AT}}} \right)/{\text{AC}}} \right) \times 100,$$

where AC is the absorbance of the control and AT is the average absorbance of the test samples.

A potential fungal culture labeled FVP4 was selected due to its ability to decolorize RR31 dye more efficiently as compared to other selected fungal cultures.

### 18S rRNA identification of potent RR31 dye decolorizing fungal culture

The fungal culture FVP4 was selected and identified based on both microscopic morphology and nucleotide sequence analysis. Using consensus primers, 18S rRNA, ITS1, 5.8S rRNA, ITS2, and 28S rRNA gene fragment were amplified using high-fidelity PCR polymerase. The genomic DNA of fungi was extracted from the cells using genomic DNA isolation kit. The forward primer (5′–3′) GGAAGTAAAAGTCGTAACAAGG and reverse primer (5′–3′) GGTCCGTGTTTCAAGACGG were used for DNA amplification. Amplification began with a DNA denaturation step for 5 min at 94 °C; the following cycles included 30 s denaturation at 94 °C, 30 s annealing at 56 °C and 1.30 s extension at 72 °C and were repeated 35 times. Primer extension at 72 °C for 10 min completed the final cycle. The PCR products were loaded on 1.0% agarose gel along with StepUpTM 500 bp DNA ladder. Amplified product was gel purified and purified products were sequenced.

### Preparation of fungal pellets

Pellets of selected indigenous *Aspergillus bombycis* culture were prepared as described by Hai et al. (2013). In 250 mL Erlenmeyer flask, 2 mL of culture suspension was inoculated in 100 mL of potato dextrose broth and incubated in an incubator shaker (120 rpm) at 30 °C for 72 h. Spherical fungal pellets having a size in the diameter range of 0.2–0.5 cm were formed. Culture media was filtered with 100 µm size sieve to separate fungal pellets from media. Further experiments were carried out using fungal pellets and were added in experimental flasks on wet weight basis.

### Flask level optimization of operational conditions for RR31 decolorization using fungal pellets

Influence of initial dye concentrations was determined using 5, 10, 15, 20, and 25 mg/L of RR31 dye. 8 g of fungal pellets was inoculated in Erlenmeyer flask (250 mL) containing 100 mL of dye containing PDB medium (6 pH). After inoculation, flask was incubated at 30 °C at 120 rpm for 12 h. After the incubation period, 2 ml aliquots were withdrawn, centrifuged at 7000 rpm for 20 min, and monitored for dye decolorization. Effect of agitation speed on dye decolorization (20 mg/L RR31) by fungal pellets was determined by inoculating PDB (6 pH) medium (inoculated with 8 g of fungal pellets) and incubated at 0 (static), 50, 100 and 150 rpm in an incubator shaker at 30 °C for 12 h. After incubation, the medium was monitored for dye decolorization. The optimal biomass concentration was determined by treating 100 mL of RR31 containing PDB (6 pH) with fungal pellets at various weights of 1.0, 2.0, 4.0, 6.0, and 8.0 g. The initial RR31 concentrations were kept constantly at 20 mg/L. After inoculation flask was incubated at 30 °C at 150 rpm for 12 h. After the incubation period, aliquots were withdrawn and monitored for dye decolorization. Effect of pH for RR31 dye decolorization by potent fungal culture was determined at 3, 4, 5, 6, 7, and 8 pH. 6 g of fungal pellets was inoculated in 250 mL of Erlenmeyer flask containing 100 mL of PDB medium (20 mg/L dye). After inoculation flask was incubated at 30 °C at 150 rpm for 12 h. After the incubation period, aliquots were withdrawn and monitored for dye decolorization. For optimal temperature determination, 100 mL of culture medium (6 pH) containing 20 mg/L of RR31 dye was inoculated with 6 g of fungal pellets and incubated at 20, 25, 30, 35, and 40 °C in an incubator shaker at 150 rpm for 12 h. After the incubation period, aliquots were withdrawn and monitored for dye decolorization.

### Enzymatic studies for potential fungal culture

#### Preparation of cell-free extract

The freshly prepared fungal pellets were activated in 250 mL Erlenmeyer flasks containing 100 mL sterile PDB medium (RR31 dye added in medium) of pH 6.0, incubated at 35 °C for 12 h under shaking (150 rpm) condition, and cells were harvested by centrifugation at 7000 rpm for 20 min. The culture supernatant obtained after centrifugation was directly used as a source of extracellular enzymes. 0 h enzyme activity was determined by culture inoculated in nutrient media without dye.

#### Enzyme assay

Azoreductase, laccase, tyrosinase, phenol oxidase, and manganese peroxidase activity of culture supernatant of fungal pellets was carried out. The activity of azoreductase enzyme was assayed by monitoring NADH disappearance at 440 nm based on the modified procedure as described by Kalyani et al. (2009). Reaction mixtures for the standard assay contained in a total volume of 2.0 mL: 50 mM phosphate buffer pH 7.4, 1 mM NADH, 0.25 mM dye solution, and 200 μL of enzyme solution. Initiation of the reaction was carried out by the addition of NADH followed by monitoring the decrease in color intensity at 536 nm. Tyrosinase activity was determined by the method of Zhang and Flurkey (1997). Laccase activity was determined in a reaction mixture of 2.3 mL, containing 1.4 mL distilled water, 0.6 mL of 0.1 M sodium acetate buffer, and 0.3 mL ABTS (5 mM). The reaction mixture was mixed with 0.3 mL of enzyme solution and incubated for 2 min. After incubation 0.3 mL of H_2_O_2_ was added and change in absorbance was measured at 420 nm. Manganese peroxidase activity was determined in a reaction mixture of 0.5 mL, containing 0.1 mL sodium lactate, 0.2 mL bovine serum albumin, 0.05 mL MnSO_4_, 0.05 mL H_2_O_2_, and 0.1 mL phenol red. The reaction mixture was mixed with 0.5 mL of enzyme solution and incubated for 5 min. After incubation, 0.04 mL of NaOH was added and change in absorbance was measured at 610 nm. For the determination of phenol oxidase activity, 0.1 mL of enzyme solution was mixed with 0.8 mL of citrate phosphate buffer and 0.01 mL of dye and incubated for 4 min. After incubation, 0.1 mL of guaiacol was added and increase in absorbance was measured. All enzyme assays were carried out at 37 °C, where the reference blanks contained all components except an enzyme. All enzyme assays were run in triplicate and average activity was calculated.

### Design and development of lab-scale fungal pelleted aerobic reactor

Like other industrial wastewater, dyestuff containing industrial effluents is produced in huge amounts with comparatively high flow rates. A reactor system with larger working volume is required for the treatment of high volumes of dye containing wastewater. Therefore, the future research should be focused not only for the development of feasible microbial process using immobilized cells but also for extensive research in bioreactor design. This helps in solving some of the engineering problems.

It is also extremely essential to produce adequate data on larger bioreactor systems to support the design engineering for the conversion of flask level results into large scale commercial realities. A lab-scale rectangular reactor of 5 L capacity (working volume: 2 L) was prepared from borosilicate glass with dimensions of 30.5 × 23 × 23 cm (L × B × H). 160 g of fungal pellets were added to the reactor. Aeration was supplied to the fungal pellets from the bottom of the reactor by the means of diffusers connected to four aquarium pumps. Tubes connected with aerator were equipped with filters for the removal of air microflora. This helps in minimization of contamination chances in aerobic reactor. The adequate mixing was provided by means of diffused air flow for keeping the culture suspended which is essential for effective biodegradation. The dissolved oxygen concentration was maintained at 5–6 mg/L. pH and temperature of the simulated effluent was maintained at 6 and 35 ± 5 °C, respectively. The pH level and temperature in the bioreactor was monitored on regular basis. The efficiency of the aerobic reactor was determined by preparing simulated effluent (two times diluted PDB amended with 20 mg/L RR31 dye). The aerobic reactor was operated at HRT of 6, 12 and 24 h. The fungal dye decolorizing potential was determined by sampling feed and effluent from the aerobic bioreactor. The treatment efficiencies of aerobic bioreactor were determined in terms of COD reduction (%), TOC reduction (%) and color removal. TOC of samples were analyzed with a multi N/C 2000 Analytic Jena analyzer. COD of decolorized samples were measured according to the procedure outlined in Standard Methods (APHA et al. 1998) to determine the extent of degradation.

Percent COD and TOC reduction of treated simulated effluent were calculated as per Eqs. (2) and (3):

2 $${\text{COD reduction }}\left( \% \right) \, = \, \left( {\left( {{\text{initial COD}} - {\text{final COD}}} \right)/{\text{initial COD}}} \right) \times 100$$

3 $${\text{TOC reduction }}\left( \% \right) \, = \, \left( {\left( {{\text{initial TOC}} - {\text{final TOC}}} \right)/{\text{initial TOC}}} \right) \times 100$$

GC–MS analysis of decolorized samples was carried using a Thermo Scientific TSQ 8000 mass spectrophotometer equipped with TSQ 8000 triple quadrupole MS detector. Gas chromatography was conducted in temperature programming mode with a TG 5MS column (30 m × 0.25 mm; 0.25 µm). The initial column temperature was 80 °C for 2 min, then increased linearly at 10 °C per minute to 280 °C and held for 10 min. The helium was used as a carrier gas; flow rate was maintained at 1 mL per minute and 26 min run time. Intermediates were identified by comparing their mass spectra with the GC–MS spectral library (NIST). Recyclability of fungal pellets in the aerobic reactor was also determined in the range of 1–6 cycles.

## Results and discussion

**Fig. 1:**
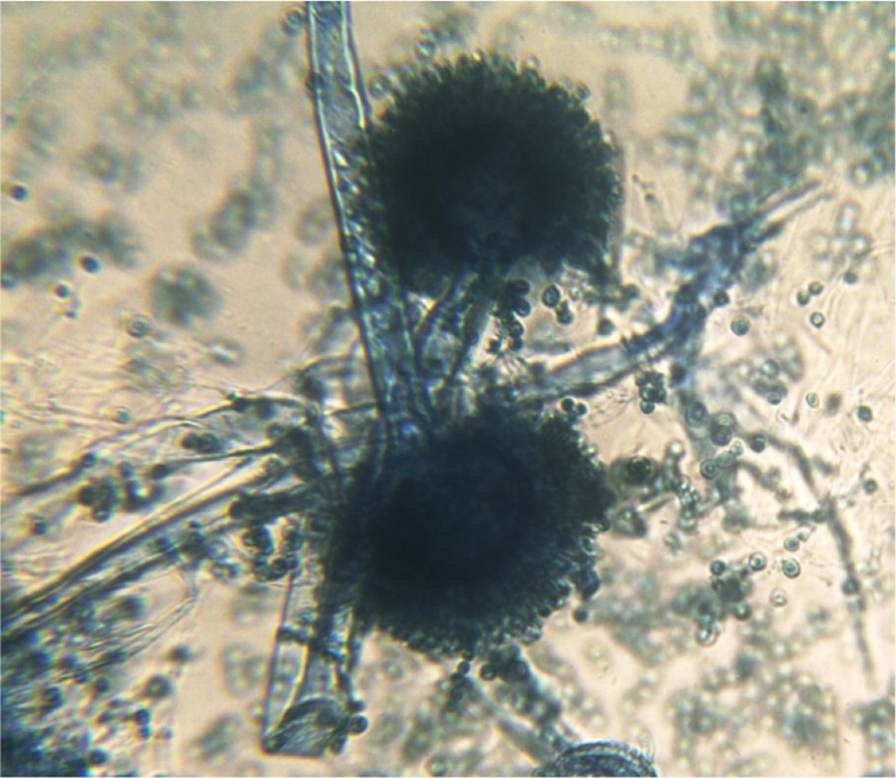
Fungal culture stained with Lactophenol cotton blue observed under ×400 magnification of light microscope

Among nine isolated and selected fungal strains, strain FVP4 gave the highest color removal efficiency (94.73% of 10 mg/L of RR31), hence, it was selected for the further studies. Morphological characteristics of potent fungal culture are shown in Fig. 1. Lactophenol cotton blue staining designated the fungal strain to be *Aspergillus* sp., conidial heads appeared radiate and large. Conidiophores were smooth-walled; the conidial heads appeared biseriate and conidia seemed globose to subglobose in shape, rough-walled, and black in color (Salar and Aneja 2007).

### 18S rRNA sequencing based identification and cultural morphology of potential fungal culture

**Fig. 2:**
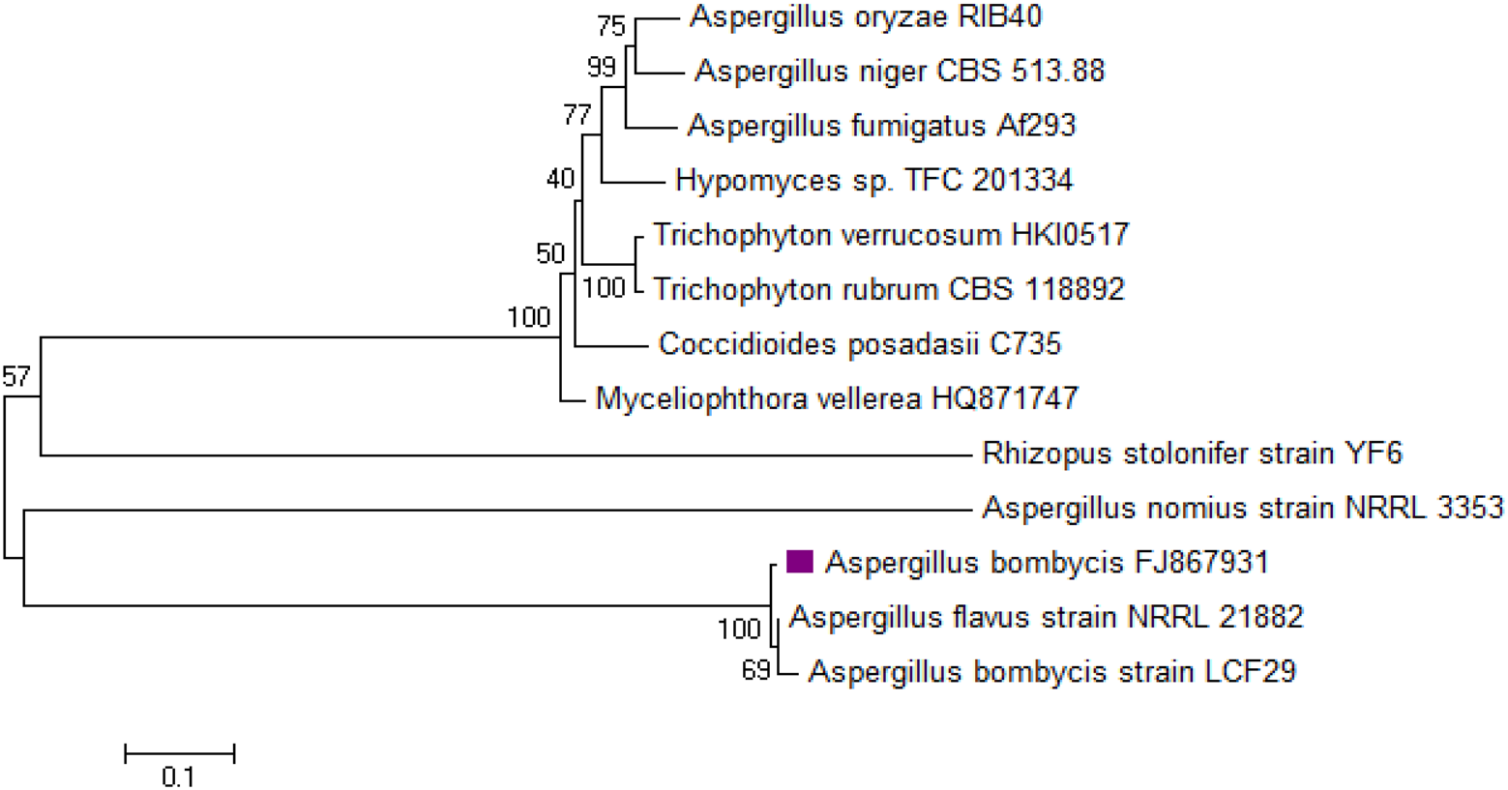
Phylogenetic tree of fungal culture was constructed using the neighbor-joining method with the aid of MEGA 6.0 program

Isolation of bacterial culture capable of the aerobic decolorization of sulfonated azo dyes has proven difficult (Srinivasan and Viraraghavan 2010), hence for the aerobic degradation of Reactive Red 31, fungal culture FVP4 isolated from dye contaminated soil was selected. The fungal strains FVP4 was further identified by analyzing the amplified gene sequence of partial 18S rRNA, ITS1, 5.8S rRNA, ITS2, and partial 28S rRNA gene. Gene sequence of fungal strain was compared with the sequence of already reported strains. A phylogenetic tree was constructed by Molecular Evolutionary Genetics Analysis (MEGA) software version 5.0 (Tamura et al. 2011). NCBI BLAST search of the sequences of the fungi FVP4 showed maximum homology with *A. bombycis*. The dendrogram (Fig. 2) reflects that isolated fungal strain FVP4 is closely related to *A. bombycis*. Hence, it was identified and designated as *A. bombycis*.

### Cultural optimization of potent fungal culture for RR31 dye decolorization

The fungal culture efficiency for dye removal could be affected by several operational conditions such as pH, temperature, concentration, and structure of the dyes, and the oxygen transfer rate (Solís et al. 2012).

**Fig. 3:**
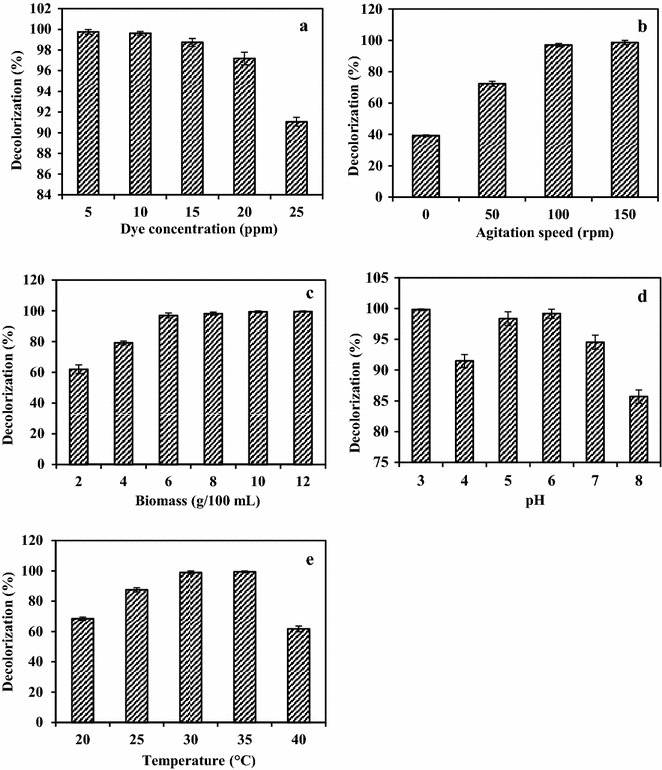
**a** Effect of initial dye concentration (pH: 6; temperature: 30 °C; agitation speed: 120 rpm; biomass concentration: 8 g), **b** agitation speed (pH: 6; temperature 30 °C; biomass concentration: 8 g; dye concentration: 20 mg/L), **c** biomass concentration (pH: 6; temperature 30 °C; dye concentration: 20 mg/L; agitation speed: 150 rpm), **d** pH (temperature 30 °C; dye concentration: 20 mg/L; agitation speed: 150 rpm; biomass concentration: 6 g) and **e** temperature (pH: 6; dye concentration: 20 mg/L; agitation speed: 150 rpm; biomass concentration: 6 g) on RR31 decolorization using potent fungal culture after 12 h of incubation period

The influence of initial dye concentration on dye decolorization has been widely reported. Effect of initial dye concentration on RR31 decolorization using fungal pellets is shown in Fig. 3a. After incubation for 12 h, decolorization decreased from 99.76 to 91.06% as dye concentration increased from 5 to 25 mg/L. The implications of high initial dye concentrations conform to most observations (Radha et al. 2005), suggesting that toxicity of dye might be an aspect that confines dye decolorization by fungal culture. This also might be due to blockage of active sites of azoreductase enzyme by dye molecules with different structures. Also, sulfonic acid (SO_3_H) groups containing reactive dyes greatly inhibited fungal growth at higher dye concentrations (Khan et al. 2013). Similar results were reported by Chen and Ting (2015) where, the decolorization efficiency of *Coriolopsis* sp. for methyl violet, crystal violet, and malachite green appeared to be better at lower initial dye concentrations (50, 100 mg/L) as compared to higher dye concentration (200 mg/L). Generally, 50 mg/L of initial dye concentration of these allowed rapid decolorization within 6 (97%), 5 (97%), 5 (98%), and 1 days (91%), respectively, while 200 mg/L required a longer duration of up to 14 days, and even then with only 7–79% decolorization efficiency.

Potent fungal culture, *A. bombycis* showed maximum RR31 dye decolorization potential (98.62%) at 150 rpm of agitation speed. Dye decolorization potential of culture was lowest (39.17%) at 0 rpm (static conditions). In this case, agitation increased mass and oxygen transfer between cells and the nutrient medium. In addition to this, enzyme activities also could have dependent on the presence of oxygen (Saratale et al. 2011). At agitation speed of 50 and 100 rpm, the culture showed 72.34 and 97.01% decolorization, respectively, after 12 h of incubation (Fig. 3b). Similar results have been reported by Chakraborty et al. (2013), where at an agitation speed of 150 and 120 rpm, the fungus *Alternaria alternata* CMERI F6 was able to decolorize 99.99% of 600 mg/L Congo Red dye. The dye removal rate was also maximal (5.59 mg/L/h) at 150 rpm showing that optimum aeration is necessary for maximal dye removal. At agitation speed of 90 and 180 rpm, the fungus showed relatively less decolorization performance, giving 93 and 89% decolorization after 48 h of incubation, respectively.

Defining the effect of fungal biomass concentration on RR31 decolorization is also an essential factor as there is a direct relationship between dye decolorization and inoculum size. Therefore, this study has been carried out to determine the effect of different inoculum size on decolorization potential. 2, 4, 6, 8, 10, and 12 g of fungal pellets were added in 100 mL of media on wet weight basis. Maximum decolorization potential (99.58%) was achieved with 12 g, followed by 10 g (99.37%) and 8 g (98.18) of fungal pellets (Fig. 3c). A minor difference in decolorization potential in case of 12 and 8 g of inoculum size was observed. Hence, for the sake of economic feasibility, 8 g of inoculum size was selected for further studies. The application of the higher amount of biomass would only be considered if the dye concentrations are higher than 100 mg/L and when rapid decolorization is required. This has been reported by Saratale et al. (2006) who demonstrated that 200 and 500 mg/L of triphenylmethane dyes were efficiently decolorized within 24 h by 10 g (wet weight) of *Aspergillus ochraceus*. In contrast, 0.5 g of *Pencillium ochrochloron* was sufficient to decolorize (93% decolorization) 50 mg/L of Cotton Blue within 2.5 h (Shedbalkar et al. 2008). The advantage of using higher biomass concentration has also been studied by other researchers (Saratale et al. 2006; Jadhav and Govindwar 2006; Shedbalkar et al. 2008; Parshetti et al. 2011; Chen and Ting 2015), attributed to availability of more number of cells to secrete extracellular enzymes responsible for biodegradation (Abedin 2008). Higher biomass concentration (6 and 8 g) also maintained metabolic functions and cell viability for a longer time period, supporting biodegradation and decolorization process (Bergsten-Torralba et al. 2009).

Adaptation of the fungal culture to a broader range of pH can make it more suitable for dye bioremediation. The potent fungal culture showed RR31 decolorization up to 99.16% in the broad range of pH (4–8). Optimum pH for RR31 decolorization was found to be 6. Removal of dye at 3 pH was found to be 99.83% (Fig. 3d), which was probably due to adsorption rather than biodegradation, as the dye was found to be adsorbed to the biomass after 12 h of incubation period. The dye removal efficiency of fungal culture makes it a potential candidate for dye removal from industrial wastewater that differs significantly in pH (Saratale et al. 2011). The effects of pH may be related to the transport of dye molecule across the cell membrane, which is considered as the rate limiting step for the dye decolorization (Khan et al. 2013). *A. alternata* CMERI F6 showed the decolorization of Congo Red up to nearly 99.99% in the pH range of 3–7. Although the fungus could effectively decolorize (89%) Congo Red at relatively higher 8 pH, it showed maximal rate (6.32 mg/L/h) of dye decolorization at pH 5 (Saratale et al. 2011).

Temperature affects the properties of the aqueous environment as well as all metabolic processes of the organism including nutrient availability and uptake. Also, reports showed that in microbial physiology, change in temperature leads to a sudden alteration in the activation energy (Khan et al. 2013). This is attributed to the fact that in degradative decolorization, the optimum temperature is necessary for the optimal activity of dye degradative enzymes (Saratale et al. 2011). Adaptation of the fungal strain to a broader range of temperature can make it a potential candidate for dye bioremediation. During optimization of cultural condition for the decolorization of RR31 dye using fungal granules, it was found that temperature has a profound influence on dye decolorization. RR31 decolorization was significantly increased (Fig. 3e) with increasing temperature, and maximum decolorization (99.37%) was observed at 35 °C for 20 mg/L dye at 6 pH after 12 h of incubation. Results indicated that, by further increasing the incubation temperature at 40 °C, a gradual decrease (61.76%) in decolorization occurred. A study carried out by Chakraborty et al. (2013) showed that 99.99% decolorization of 600 mg/L Congo Red was observed at 20, 25, and 30 °C within 44, 40, and 48 h, respectively.

### Enzymatic studies

Fungi possess a highly versatile enzymatic system capable of acting and efficiently degrading recalcitrant azo dye molecules. A wide range of oxidoreductase enzymes is involved in decolorization process and they act by biotransforming the parent dye molecule into compounds having lesser toxicity (Pandey et al. 2007). The major mechanism behind biodegradation of dyes using fungal cultures is their ability to produce enzymes such as laccase, manganese peroxidase, azoreductase, tyrosinase, and phenol oxidase. According to previous reports, these enzymes might be responsible for dye degradation (Khan et al. 2013). The unspecific activity of enzymes is the first step in the process of mineralization of reactive dyes (Raghukumar et al. 1996). The relative contributions of these enzymes for the decolorization of dyes may be different for each fungal culture.

**Fig. 4:**
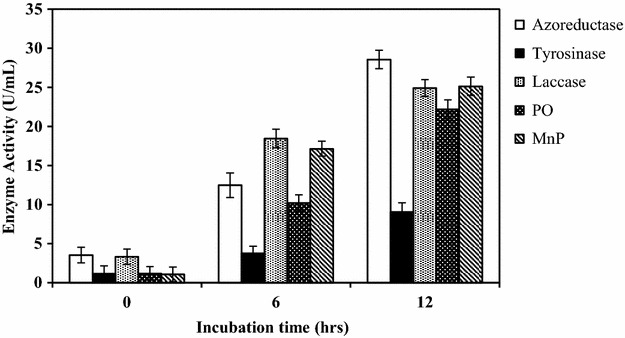
Azoreductase, tyrosinase, laccase, phenol oxidase, and manganese peroxidase activity in initial to final stage of Reactive Red 31 decolorization at 35 °C

In the present study, a significant increase in enzyme activity of azoreductase, laccase, phenol oxidase, and manganese peroxidase was observed after Reactive Red 31 decolorization by fungal culture FVP4 (Fig. 4). The activity of azoreductase, laccase, phenol oxidase, and manganese peroxidase in fungal culture after decolorization (28.57, 24.92, 22.19, and 25.14 U/mL, respectively) was about 8, 7.5, 19, and 23.7 fold more than before decolorization (3.53, 3.31, 1.17, and 1.06 U/mL, respectively) suggesting that these enzymes might be induced by the addition of Reactive Red 31 dye, and thus results in a higher decolorization.

Similar results were reported by Sinha and Osborne (2016), where the activity of laccase was observed to be significantly higher in the decolorized broth obtained after the decolorization of Reactive Green by *Aspergillus* sp. Higher azoreductase activity in shaking condition is attributed to variation in azoreductase types produced by few microbial strains (Ali et al. 2009a, b). The MnP and Laccase activities in *C. globosum* were significantly favored by agitation with maximum values of 250 and 83.4 U/L, respectively, during remediation of dye containing effluent (Manai et al. 2016).

### Performance of lab-scale fungal pelleted aerobic reactor for the remediation of RR31 dye

The application of fungal pellets in bioreactors has fascinated environmental bioengineers due to their potential in continuous and semi-continuous system operation. As well as their capacity to diminish operational difficulties instigated by fungal disperse mycelium (Espinosa-Ortiz et al. 2016). Immobilized fungal biological reactors have been found to display good bio-activities for longer time duration (Singh 2006). A wide variety of fungal cultures including *Aspergillus* sp. is capable of removal of a wide range of dyes (Fu and Viraraghavan 2001). Many fungal genera have been employed either in living or inactivated form.

**Fig. 5:**
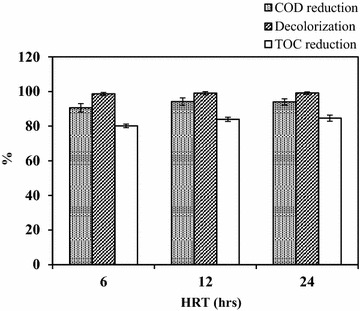
Performance of designed and developed aerobic reactor in terms of decolorization, TOC, and COD reduction

The results showed that highest dye decolorization potential was achieved at 12 h (99.02%) and 24 h (99.09%) of HRT in the aerobic bioreactor. The highest percentage of COD reduction was achieved in 12 h (94.19%) followed by 24 h (93.97%) of HRT. Percentage of TOC reduction in 6, 12 and 24 h of HRT was found to be 80.14, 83.97 and 84.62%, respectively (Fig. 5). 91–99% removal of 100 mg/L of Reactive Red 198, Reactive Black 5 and Reactive Blue 19 was observed in stirred tank reactor using pellets of *Coriolus versicolor* (Borchert and Libra 2001). In an airlift column reactor using pellets of *P. oxalicum*, complete removal of Cu-complex Reactive Blue 21 dye (200 mg/L) was achieved in 12 h (Xin et al. 2010). A bubble column reactor with pellets of *P. ostreatus* was also proved as a suitable alternative for the cotton pulp liquor decolorization (Zhao et al. 2008). The recycling of the fungal pellets supported the maximal color removal efficiency of 76% from the effluent. The system was operated in repeated batch modes, for four cycles. The COD removal was found to be around 70%. Isolated fungal strain *A. bombycis* showed better dye decolorization performance in short duration of time (12 h) as compared to other reported fungal cultures (Table 1).

**Table 1:** Comparative performance of selected *Aspergillus bombycis* culture with other fungal strains for dye removal from aqueous solutions

Fungal strain	Dye (concentration)	Time duration	Decolorization (%)	References
*Aspergillus niger*	Congo Red (10 mg/L)	36 h	99	Karthikeyan et al. (2010)
*Aspergillus niger*	Acid Red 151 (20 mg/L) Orange II (20 mg/L)	24 h	98 84	Ali et al. (2009a, b)
*Aspergillus bombycis*	Reactive Red 31 (20 mg/L)	12 h	99	Present study
*Armillaria* sp.	Reactive Black 5 (100 mg/L)	96 h	65	Hadibarata et al. (2012)
*Phanerochaete chrysosporium*	Direct Red 80 (20 mg/L)	72 h	100	Sen et al. (2012)
*Trametes versicolor*	Blue 49 (50 mg/L)	7 days	94	Pilatin and Kunduhoglu (2011)
*Pleurotus sajor*-*caju*	Reactive Blue 220 (50 mg/L)	11 days	100	Munari et al. (2008)
*Phanerochaete chrysosporium*	Direct Red 80 (50 mg/L)	24 h	100	Singh et al. (2010)

**Fig. 6:**
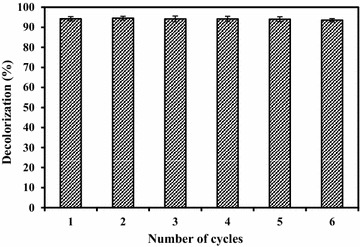
Recycling of fungal pellets in aerobic bioreactor at 12 h of HRT

For the application in environmental engineering, reutilization of fungal pellets could be both technically demanding and biologically challenging considering the amount of residual dye that is concentrated (in adsorptive decolorization) when recycling the pellets. Experiments on recycling of fungal pellets in aerobic bioreactor were carried out to determine its reusability. The recycle of used pellets is hardly exploited under the tested conditions. From the results, it is clear that more than 94% decolorization was observed up to 5th cycle (Fig. 6). The increase in decolorization efficiency (94.51%) in the 2nd cycle may be attributed to acclimatization of fungal pellets towards RR31 dye. Similar results were reported by Kaushik et al. (2014), where, *Aspergillus lentulus* successfully decolorized 90% of 100 mg/L of Acid Blue 120 up to 5 reused cycles in the fungal pelleted aerobic bioreactor.

**Fig. 7:**
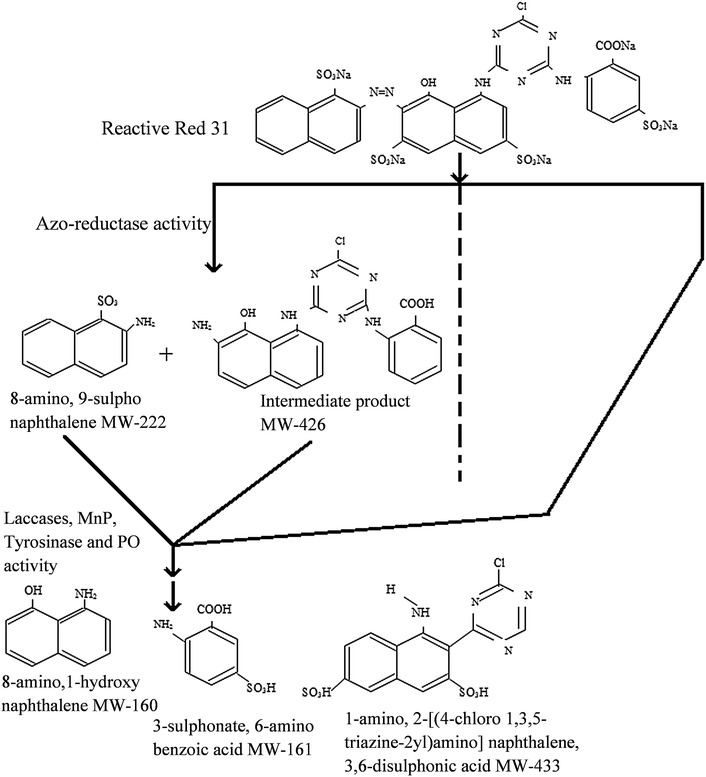
Proposed biodegradation pathway of Reactive Red 31 by *Aspergillus bombycis* pellets

GC–MS analysis was carried out to determine the intermediates formed during degradation of RR31 by fungal culture in aerobic reactor system (Fig. 7). GC–MS analysis of metabolites extracted at different time intervals from the aerobic reactor indicated formation of some intermediated product such as 8-amino-1-hydroxynaphthalene, 3-sulfonate-6-amino benzoic acid, 1-amino-2-[(4-chloro-1,3,5-triazine-2yl)amino] naphthalene-3,6-disulfonic acid, 8-amino 9-sulphonaphthalene, and one intermediated product after 12 h of incubation due to asymmetric/symmetric cleavage of the RR31 by ligninolytic enzymes and other oxidoreductases with a mass peak at 161, 217, 433 and 426, respectively.

## Conclusions

Fungal-based techniques play an important role in degradative removal of sulfonated azo dyes. The current study focuses on the dye remediation potential of *A. bombycis* pellets. The RR31 dye decolorization potential of isolated *A. bombycis* culture was influenced by initial dye concentration, biomass concentration, pH, temperature, and required aerated conditions. Biodegradation (induction of azoreductase, laccase, phenol oxidase, and Mn-peroxidase activities) played an essential role in dye decolorization, which resulted in reduced absorbance for dyes. At 12 h of HRT in a developed fungal pelleted aerobic bioreactor, decolorization efficiency, COD, and TOC reduction of 99.02, 94.19, and 83.97% was achieved, respectively. GC–MS analysis of treated effluent using developed fungal bioreactor proved biodegradation of Reactive Red 31 dye. *Aspergillus bombycis* pellets showed potential for the application of dye bioremediation in industrial wastewater. However, application of selected fungal culture for the degradation of higher concentration of Reactive Red 31 dye as well as reactive dye mixture is still in the research stage. Efforts are required for the commercialization of current research through: (a) pilot scale studies with real industrial effluent and (b) system involving biosorbents and immobilized enzyme systems.

## References

[CR1] Abd El-Rahim WM, Mostafa EM, Moawad H (2016). High cell density cultivation of six fungal strains efficient in azo dye bioremediation. Biotechnol Rep.

[CR2] Abedin MAR (2008). Decolorization and biodegradation of crystal violet and malachite green by *Fusarium solani* Martius Saccardo, a comparative study on biosorption of dyes by the dead fungal biomass. Am Euras J Bot.

[CR3] Ali N, Hameed A, Ahmed S (2009). Physicochemical characterization and bioremediation perspective of textile effluent, dyes and metals by indigenous bacteria. J Hazard Mater.

[CR4] Ali N, Hameed A, Siddiqui MF, Ghumro PB, Ahmed S (2009). Application of *Aspergillus niger* SA1 for the enhanced bioremoval of azo dyes in simulated textile effluent. Afr J Biotechnol.

[CR5] APHA, AWWA, WPCF (1998) Standard methods for the examination of water and wastewater, 20th edn. American Public Health Association, Washington, DC

[CR6] Bergsten-Torralba LR, Nishikawa MM, Baptista DF, Magalhaes DP, Da Silva M (2009). Decolorization of different textile dyes by *Penicillium simplicissimum* and toxicity evaluation after fungal treatment. Braz J Microbiol.

[CR7] Borchert M, Libra JA (2001). Decolorization of reactive dyes by the white rot fungus *Trameters versicolor* in sequencing batch reactors. Biotechnol Bioeng.

[CR8] Chakraborty S, Basak B, Dutta S, Bhunia B, Dey A (2013). Decolorization and biodegradation of Congo Red dye by a novel white rot fungus *Alternaria alternata* CMERI F6. Bioresour Technol.

[CR9] Chen SH, Ting ASY (2015). Biodecolorization and biodegradation potential of recalcitrant triphenylmethane dyes by *Coriolopsis* sp. isolated from compost. J Environ Manag.

[CR10] Espinosa-Ortiz EJ, Rene ER, Pakshirajan K, Van Hullebusch ED, Lens PNL (2016). Fungal pelleted reactors in wastewater treatment: applications and perspectives. Chem Eng J.

[CR11] Fewson CA (1988). Biodegradation of xenobiotic and other persistent compounds: the causes of recalcitrance. Trends Biotechnol.

[CR12] Fu Y, Viraraghavan T (2001). Removal of acid blue 29 from an aqueous solution by *Aspergillus niger*. Am Assoc Text Chem Color Rev.

[CR13] Gomi N, Yoshida S, Matsumoto K, Okudomi M, Konno H, Hisabori T, Sugano Y (2011). Degradation of the synthetic dye amaranth by the fungus *Bjerkandera adusta* Dec 1: inference of the degradation pathway from an analysis of decolorized products. Biodegradation.

[CR14] Hadibarata T, Yusoff A, Aris A, Salmiati S, Hidayat T, Kristanti R (2012). Decolorization of azo triphenylmethane and anthraquinone dyes by laccase of a newly isolated *Armillaria* sp. F022. Water Air Soil Pollut.

[CR15] Hai FI, Yamamoto K, Nakajima F, Fukushi K, Nghiem LD, Price WE, Jin B (2013). Degradation of azo dye acid orange 7 in a membrane bioreactor by pellets and attached growth of *Coriolus versicolour*. Bioresour Technol.

[CR16] Jadhav JP, Govindwar SP (2006). Biotransformation of malachite green by *Saccharomyces cerevisiae* MTCC 463. Yeast.

[CR17] Jin XC, Liu GQ, Xu ZH, Tao WY (2007). Decolorization of a dye industry effluent by *Aspergillus fumigatus* XC6. Appl Microbiol Biotechnol.

[CR18] Kalyani DC, Telke AA, Govindwar SP, Jadhav JP (2009). Biodegradation and detoxification of reactive textile dye by isolated *Pseudomonas* sp. SUK1. Water Environ Res.

[CR19] Karthikeyan K, Nanthakumar K, Shanthi K, Lakshmanaperumalsamy P (2010). Response surface methodology for optimization of culture conditions for dye decolorization by a fungus *Aspergillus niger* HM11 isolated from dye affected soil. Iran J Microbiol.

[CR20] Kaushik R, Grochowska KM, Butnaru I, Kreutz MR (2014). Protein trafficking from synapse to nucleus in control of activity-dependent gene expression. Neuroscience.

[CR21] Khan R, Bhawana P, Fulekar MH (2013). Microbial decolorization and degradation of synthetic dyes: a review. Rev Environ Sci Biotechnol.

[CR22] Khan R, Khan Z, Bhatt N, Devecha J, Madamwar D (2014). Azo dye decolorization under microaerophilic conditions by a bacterial mixture isolated from anthropogenic dye-contaminated soil. Bioremediat J.

[CR23] Lang W, Sirisansaneeyakul S, Ngiwsara L, Mendes S, Martins LO, Okuyama M, Kimura A (2013). Characterization of a new oxygen-insensitive azo reductase from *Brevibacillus laterosporus* TISTR1911: toward dye decolorization using a packed-bed metal affinity reactor. Bioresour Technol.

[CR24] Manai I, Miladi B, Mselmi AE, Smaali I, Hassen A, Hamd M, Bouallagui H (2016). Industrial textile effluent decolourization in stirred and static batch cultures of a new fungal strain *Chaetomium globosum* IMA1 KJ472923. J Environ Manag.

[CR25] Munari FM, Gaio TA, Calloni R, Dillon AJP (2008). Decolorization of textile dyes by enzymatic extract and submerged cultures of *Pleurotus sajor-caju*. World J Microbiol Biotechnol.

[CR26] Nilsson I, Moller A, Mattiasson B, Rubindamayugi MST, Welander U (2006). Decolorization of synthetic and real textile wastewater by the use of white-rot fungi. Enzyme Microb Technol.

[CR27] Pandey A, Singh P, Iyengar L (2007). Bacterial decolorization and degradation of azo dyes. Int Biodeterior Biodegrad.

[CR28] Parshetti GK, Parshetti SG, Telke AA, Kalyani DC, Doong RA, Govindwar SP (2011). Biodegradation of crystal violet by *Agrobacterium radiobacter*. J Environ Sci.

[CR29] Pilatin S, Kunduhoglu B (2011). Decolorization of textile dyes by newly isolated *Trametes versicolor* strain. Life Sci Biotechnol.

[CR30] Radha KV, Regupathi I, Arunagiri A, Murugesan T (2005). Decolorization studies of synthetic dyes using *Phanerochaete chrysosporium* and their kinetics. Process Biochem.

[CR31] Raghukumar C, Chandramohan D, Michel F, Reddy CA (1996). Degradation of lignin and decolourisation of paper mill bleach plant effluent (BPE) by marine fungi. Biotech Lett.

[CR32] Salar RK, Aneja KR (2007). Thermophilic fungi: taxonomy and biogeography. J Agric Technol.

[CR33] Saratale GD, Kalme SD, Govindwar SP (2006). Decolorization of textile dyes by *Aspergillus ochraceus* (NCIM-1146). Indian J Biotechnol.

[CR34] Saratale GD, Saratale RG, Chang JS, Govindwar SP (2011). Fixed-bed decolorization of Reactive Blue 172 by *Proteus vulgaris* NCIM-2027 immobilized on Luffa cylindrica sponge. Int Biodeterior Biodegrad.

[CR35] Saratale RG, Gandhi SS, Purankar MV, Kurade MB, Govindwar SP, Oh SE, Saratale GD (2013). Decolorization and detoxification of sulfonated azo dye C.I. Remazol Red and textile effluent by isolated *Lysinibacillus* sp. RGS J Biosci Bioeng.

[CR36] Sen K, Pakshirajan K, Santra SB (2012). Modelling the biomass growth and enzyme secretion by the white rot fungus *Phanerochaete chrysosporium* in presence of a toxic pollutant. J Environ Prot.

[CR37] Shedbalkar U, Dhanve R, Jadhav J (2008). Biodegradation of triphenylmethane dye cotton blue by *Penicillium ochrochloron* MTCC 517. J Hazard Mater.

[CR38] Singh H (2006). Mycoremediation: fungal bioremediation.

[CR39] Singh S, Pakshirajan K, Daverey A (2010). Enhanced decolourization of Direct Red-80 dye by the white rot fungus *Phanerochaete chrysosporium* employing sequential design of experiments. Biodegradation.

[CR40] Sinha A, Osborne WJ (2016). Biodegradation of reactive green dye (RGD) by indigenous fungal strain VITAF-1. Int Biodeterior Biodegrad.

[CR41] Solís M, Solís A, Pérez H, Manjarrez N, Flores M (2012). Microbial decoloration of azo dyes: a review. Process Biochem.

[CR42] Srinivasan A, Viraraghavan T (2010). Decolorization of dye wastewaters by biosorbents: a review. J Environ Manag.

[CR43] Tamura K, Peterson D, Peterson N, Stecher G, Nei M, Kumar S (2011). MEGA5: molecular evolutionary genetics analysis using maximum likelihood, evolutionary distance, and maximum parsimony methods. Mol Biol Evol.

[CR44] Tang W, Jia R, Zhang D (2011). Decolorization and degradation of synthetic dyes by *Schizophyllum* sp. F17 in a novel system. Desalination.

[CR45] Xin B, Chen G, Zheng W (2010). Bioaccumulation of Cu-complex reactive dye by growing pellets of *Penicillium oxalicum* and its mechanism. Water Res.

[CR46] Zhang X, Flurkey WH (1997). Phenoloxidases in Portabella mushrooms. J Food Sci.

[CR47] Zhao LH, Zhou JT, Zheng CL, Yang YS, Sun HJ, Zhang XH (2008). Decolorization of cotton pulp black liquor by *Pleurotus ostreatus* in a bubble-column reactor. Bull Environ Contam Toxicol.

